# Bis[*trans*-di­fluorido­tetra­kis­(pyridine-κ*N*)chromium(III)] sodium tetra­chlorido­zincate perchlorate from synchrotron data

**DOI:** 10.1107/S1600536814014408

**Published:** 2014-06-25

**Authors:** Dohyun Moon, Keon Sang Ryoo, Jong-Ha Choi

**Affiliations:** a Pohang Accelerator Laboratory, POSTECH, Pohang 790-784, Republic of Korea; b Department of Chemistry, Andong National University, Andong 760-749, Republic of Korea

**Keywords:** crystal structure

## Abstract

The title salt, Na[CrF_2_(C_5_H_5_N)_4_]_2_[ZnCl_4_]ClO_4_, consists of two cationic Cr^III^ complexes, an Na^+^ cation, one [ZnCl_4_]^2−^ anion and one ClO_4_^−^ anion. The Cr^III^ atoms are coordinated by four pyridine (py) N atoms and two F atoms in a *trans* arrangement, displaying a distorted octa­hedral geometry. The mean Cr—N(py) and Cr—F bond lengths are 2.086 (8) and 1.864 (14) Å, respectively. The [ZnCl_4_]^2−^ anion has a distorted tetra­hedral geometry. The most notable feature of the crystal packing is the formation of weak pyridine–perchlorate C—H⋯O hydrogen bonds, resulting in supra­molecular chains along the *b-*axis direction. The perchlorate anion was disordered over two sets of sites in a 0.868 (3):0.132 (3) ratio.

## Related literature   

For the synthesis of *trans*-[Cr(py)_4_F_2_]NO_3_, see: Glerup *et al.* (1970 ▶). For the structures of *trans*-[Cr(py)_4_F_2_]ClO_4_ and *trans*-[Cr(py)_4_F_2_]PF_6_, see: Moon & Choi (2013 ▶); Fochi *et al.* (1991 ▶). Chromium(III)-doped crystals are promising materials for tunable solid state lasers in the spectral region between 600 and 1100 nm, see: Powell (1998 ▶).
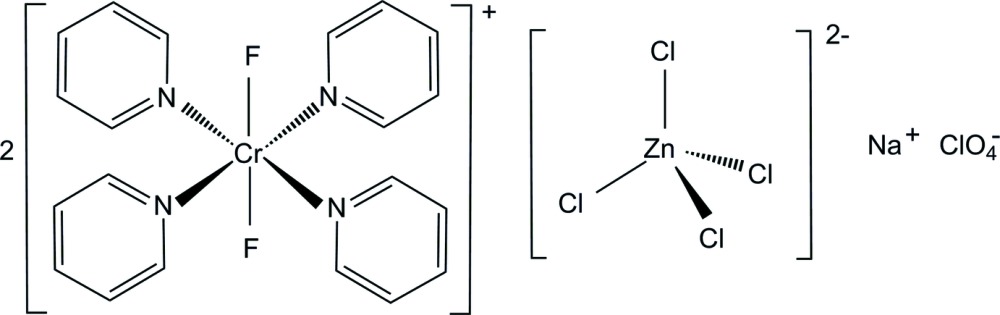


## Experimental   

### 

#### Crystal data  

Na[CrF_2_(C_5_H_5_N)_4_]_2_[ZnCl_4_]ClO_4_
*M* *_r_* = 1142.41
Orthorhombic, 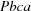

*a* = 25.397 (5) Å
*b* = 14.600 (3) Å
*c* = 25.510 (5) Å
*V* = 9459 (3) Å^3^
*Z* = 8
Synchrotron radiation
λ = 0.62998 Å
μ = 0.94 mm^−1^
*T* = 100 K
0.20 × 0.15 × 0.10 mm

#### Data collection  

ADSC Q210 CCD area-detector diffractometer
Absorption correction: empirical (*HKL3000sm*; Otwinowski & Minor, 1997 ▶) *T*_min_ = 0.835, *T*_max_ = 0.912
86381 measured reflections
13152 independent reflections
12355 reflections with *I* > 2σ(*I*)
*R*_int_ = 0.050

#### Refinement  

*R*[*F* ^2^ > 2σ(*F* ^2^)] = 0.047
*wR*(*F* ^2^) = 0.136
*S* = 1.05
13152 reflections
605 parameters
6 restraints
H-atom parameters constrained
Δρ_max_ = 1.25 e Å^−3^
Δρ_min_ = −1.99 e Å^−3^

### 

Data collection: *PAL ADSC Quantum-210 ADX* (Arvai & Nielsen, 1983 ▶); cell refinement: *HKL3000sm* (Otwinowski & Minor, 1997 ▶); data reduction: *HKL3000sm*; program(s) used to solve structure: *XS* in *SHELXS2014* (Sheldrick, 2008 ▶); program(s) used to refine structure: *XL* in *SHELXL2014* (Sheldrick, 2008 ▶); molecular graphics: *DIAMOND* (Brandenburg, 2007 ▶); software used to prepare material for publication: *WinGX* (Farrugia, 2012 ▶).

## Supplementary Material

Crystal structure: contains datablock(s) I, 70. DOI: 10.1107/S1600536814014408/tk5321sup1.cif

Structure factors: contains datablock(s) I. DOI: 10.1107/S1600536814014408/tk5321Isup2.hkl

CCDC reference: 1009068

Additional supporting information:  crystallographic information; 3D view; checkCIF report

## Figures and Tables

**Table 1:** Hydrogen-bond geometry (Å, °)

*D*—H⋯*A*	*D*—H	H⋯*A*	*D*⋯*A*	*D*—H⋯*A*
C2—H2⋯O5*P* ^i^	0.95	2.31	3.237 (14)	165
C10—H10⋯O2*P* ^ii^	0.95	2.49	3.352 (3)	151

Symmetry codes: (i) 
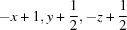
; (ii) 
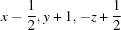
.

**Fig. 1.**
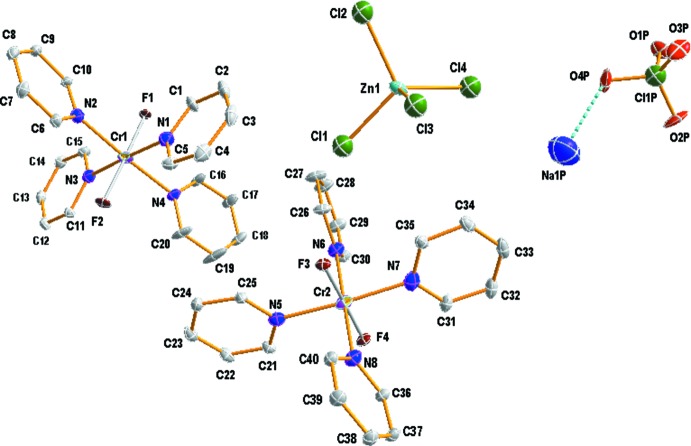
A perspective view of the molecular structures of the asymmetric unit in the title compound

## supplementary crystallographic information

### S1. Structural commentary

The preparation and characterization of novel chromium(III) systems have received much attention because chromium(III) doped crystals are promising materials for tunable solid state laser in the spectral region between 600 and 1100 nm (Powell, 1998). Anionic species also play a very important role in chemistry, medicine and in biology, yet their binding characteristics have not received much recognition. The study of the anion effect and geometrical isomer in o­cta­hedral transition metal complexes may be expected to yield a great variety of new structures and properties of both chemical and biological significance. The [Cr(py)_4_*X*_2_]^n+^(*X* = monodentate; py = pyridine) cation can be either *trans* or *cis* geometric isomers.

In this communication, we report the synthesis and structure of 2[Cr(C_5_H_5_N)_4_F_2_]^+^ [ZnCl_4_]^2-^ Na^+^ ClO_4_^-^ in order to confirm the arrangement of four py molecules and two F ligands in the complex. This is another example of a *trans*-[Cr(py)_4_F_2_]^+^ complex but with a different counter anion (Fochi *et al.*, 1991; Moon & Choi, 2013). The asymmetric unit of the title salt contains discrete two *trans*-[Cr(py)_4_F_2_]^+^, one [ZnCl_4_]^2-^, one Na cation and one disordered ClO_4_^-^ anion. The structural analysis shows that there are two independent Cr(III) complex cations in which the four nitro­gen atoms of four py ligands occupy the equatorial sites and the two F atoms coordinate to the Cr atom in *trans* configuration. An ellipsoid plot (60% probability level) of the title compound, together with the atomic labelling, is depicted in Fig. 1.

The Cr—N(py) bond distances varies from 2.0759 (18) to 2.0986 (18) Å and the Cr–F bond distances are in the range of 1.8532 (12) to 1.8838 (12) Å. These bond lengths are in good agreement with those observed in *trans*-[Cr(py)_4_F_2_]PF_6_ and *trans*-[Cr(py)_4_F_2_]ClO_4_.

The uncoordinated [ZnCl_4_]^2-^ anion remains outside the coordination sphere. As expected, the Zn atom in the [ZnCl_4_]^2-^ has a distorted tetra­hedral geometry surrounded by four Cl atoms. The Zn—Cl bond distance of 2.0228 (19)–2.5558 (7) Å and the Cl—Zn—Cl angles of 100.05 (6)–121.91 (3)° are observed, respectively. The ClO_4_^-^ also has distorted tetra­hedral geometry due to the influence of hydrogen bonding and connecting Na^+^ ion on the Cl—O lengths and the O—Cl–O angles. In the title complex, the crystal lattice is stabilized by weak hydrogen bonding inter­actions between the C—H groups of the py ligand and the O atoms of the ClO_4_ anion (Table 1).

### S2. Synthesis and crystallization

All chemicals were reagent grade materials and used without further purification. As starting material, *trans*-[Cr(py)_4_F_2_]NO_3_ was prepared as described previously (Glerup *et al.*, 1970). The crude [Cr(py)_4_F_2_]NO_3_ (0.2 g) was dissolved in 10 ml water at 60 °C. The 10 ml solution of 1*M* HCl containing 0.1 g sodium perchlorate and 0.5 g of ZnCl_2_ was gradually added. The unreacted materials were removed by filtration and allowed to stand at room temperature for several days to give purple crystals of the title compound suitable for X-ray structural analysis.

### S3. Refinement

C-bound H-atoms were placed in calculated positions (C—H = 0.95) and were included in the refinement in the riding model approximation with *U*_iso_(H) set to 1.2*U*_eq_(C). The perchlorate anion was distorted over two positions in a 0.868 (3):0.132 (3) ratio.

### Crystal data

**Table:**

Na[CrF_2_(C_5_H_5_N)_4_]_2_[ZnCl_4_]ClO_4_	*D*_x_ = 1.604 Mg m^−^^3^
*M**_r_* = 1142.41	Synchrotron radiation, λ = 0.62998 Å
Orthorhombic, *P**b**c**a*	Cell parameters from 237022 reflections
*a* = 25.397 (5) Å	θ = 0.4–33.6°
*b* = 14.600 (3) Å	µ = 0.94 mm^−^^1^
*c* = 25.510 (5) Å	*T* = 100 K
*V* = 9459 (3) Å^3^	Block, purple
*Z* = 8	0.20 × 0.15 × 0.10 mm
*F*(000) = 4624	

### Data collection

**Table:**

ADSC Q210 CCD area-detector diffractometer	12355 reflections with *I* > 2σ(*I*)
Radiation source: PLSII 2D bending magnet	*R*_int_ = 0.050
ω scan	θ_max_ = 26.0°, θ_min_ = 2.0°
Absorption correction: empirical (using intensity measurements) (*HKL3000sm*; Otwinowski & Minor, 1997)	*h* = −35→35
*T*_min_ = 0.835, *T*_max_ = 0.912	*k* = −19→19
86381 measured reflections	*l* = −35→35
13152 independent reflections	

### Refinement

**Table:**

Refinement on *F*^2^	6 restraints
Least-squares matrix: full	Hydrogen site location: inferred from neighbouring sites
*R*[*F*^2^ > 2σ(*F*^2^)] = 0.047	H-atom parameters constrained
*wR*(*F*^2^) = 0.136	*w* = 1/[σ^2^(*F*_o_^2^) + (0.0773*P*)^2^ + 19.7848*P*] where *P* = (*F*_o_^2^ + 2*F*_c_^2^)/3
*S* = 1.05	(Δ/σ)_max_ = 0.002
13152 reflections	Δρ_max_ = 1.25 e Å^−^^3^
605 parameters	Δρ_min_ = −1.99 e Å^−^^3^

### Special details

**Table:**

Geometry. All e.s.d.'s (except the e.s.d. in the dihedral angle between two l.s. planes) are estimated using the full covariance matrix. The cell e.s.d.'s are taken into account individually in the estimation of e.s.d.'s in distances, angles and torsion angles; correlations between e.s.d.'s in cell parameters are only used when they are defined by crystal symmetry. An approximate (isotropic) treatment of cell e.s.d.'s is used for estimating e.s.d.'s involving l.s. planes.

### Fractional atomic coordinates and isotropic or equivalent isotropic displacement parameters (Å^2^)

**Table:**

	*x*	*y*	*z*	*U*_iso_*/*U*_eq_	Occ. (<1)
Cr1	0.35117 (2)	0.76013 (2)	0.12316 (2)	0.00754 (8)	
F1	0.30651 (4)	0.70701 (9)	0.17189 (5)	0.0118 (2)	
F2	0.39507 (5)	0.81430 (9)	0.07234 (5)	0.0134 (2)	
N1	0.37558 (7)	0.63065 (12)	0.09803 (7)	0.0116 (3)	
N2	0.28923 (7)	0.75603 (12)	0.06915 (6)	0.0105 (3)	
N3	0.32920 (6)	0.89005 (12)	0.14979 (6)	0.0099 (3)	
N4	0.41194 (6)	0.75688 (12)	0.17755 (7)	0.0107 (3)	
C1	0.35464 (10)	0.55497 (16)	0.12003 (9)	0.0188 (4)	
H1	0.3272	0.5618	0.1451	0.023*	
C2	0.37167 (12)	0.46750 (17)	0.10742 (10)	0.0258 (5)	
H2	0.3556	0.4154	0.1230	0.031*	
C3	0.41235 (11)	0.45702 (18)	0.07181 (10)	0.0268 (5)	
H3	0.4258	0.3980	0.0637	0.032*	
C4	0.43310 (10)	0.53502 (19)	0.04820 (11)	0.0252 (5)	
H4	0.4603	0.5298	0.0228	0.030*	
C5	0.41370 (9)	0.62005 (17)	0.06204 (9)	0.0180 (4)	
H5	0.4278	0.6729	0.0455	0.022*	
C6	0.29780 (8)	0.75294 (15)	0.01708 (8)	0.0143 (4)	
H6	0.3330	0.7565	0.0045	0.017*	
C7	0.25712 (9)	0.74470 (17)	−0.01887 (8)	0.0188 (4)	
H7	0.2644	0.7426	−0.0554	0.023*	
C8	0.20558 (9)	0.73955 (16)	−0.00069 (9)	0.0183 (4)	
H8	0.1771	0.7332	−0.0246	0.022*	
C9	0.19644 (8)	0.74378 (16)	0.05288 (9)	0.0165 (4)	
H9	0.1616	0.7412	0.0663	0.020*	
C10	0.23912 (8)	0.75180 (15)	0.08642 (8)	0.0133 (4)	
H10	0.2328	0.7544	0.1231	0.016*	
C11	0.35537 (8)	0.96612 (14)	0.13505 (8)	0.0125 (3)	
H11	0.3854	0.9599	0.1132	0.015*	
C12	0.33995 (9)	1.05350 (15)	0.15071 (8)	0.0165 (4)	
H12	0.3592	1.1058	0.1397	0.020*	
C13	0.29607 (9)	1.06300 (16)	0.18269 (9)	0.0177 (4)	
H13	0.2845	1.1219	0.1935	0.021*	
C14	0.26955 (9)	0.98475 (16)	0.19856 (9)	0.0173 (4)	
H14	0.2395	0.9892	0.2206	0.021*	
C15	0.28738 (8)	0.89998 (15)	0.18182 (8)	0.0137 (4)	
H15	0.2694	0.8466	0.1934	0.016*	
C16	0.40251 (9)	0.7286 (2)	0.22661 (9)	0.0259 (5)	
H16	0.3673	0.7145	0.2362	0.031*	
C17	0.44161 (10)	0.7191 (2)	0.26391 (10)	0.0288 (6)	
H17	0.4332	0.6992	0.2984	0.035*	
C18	0.49271 (9)	0.7387 (2)	0.25044 (10)	0.0245 (5)	
H18	0.5204	0.7316	0.2751	0.029*	
C19	0.50286 (10)	0.7688 (3)	0.20023 (11)	0.0441 (9)	
H19	0.5378	0.7838	0.1899	0.053*	
C20	0.46157 (9)	0.7770 (2)	0.16487 (9)	0.0301 (6)	
H20	0.4689	0.7977	0.1303	0.036*	
Cr2	0.65746 (2)	0.50377 (2)	0.11947 (2)	0.00778 (8)	
F3	0.61676 (5)	0.45700 (9)	0.06534 (4)	0.0118 (2)	
F4	0.70069 (5)	0.55143 (9)	0.17181 (5)	0.0130 (2)	
N5	0.63053 (6)	0.63505 (12)	0.10134 (7)	0.0103 (3)	
N6	0.59384 (6)	0.49421 (12)	0.17052 (6)	0.0098 (3)	
N7	0.67945 (7)	0.37247 (12)	0.14187 (7)	0.0126 (3)	
N8	0.72058 (7)	0.50992 (12)	0.06649 (6)	0.0104 (3)	
C21	0.64820 (9)	0.70809 (15)	0.12802 (8)	0.0143 (4)	
H21	0.6759	0.6999	0.1526	0.017*	
C22	0.62733 (10)	0.79550 (16)	0.12082 (9)	0.0187 (4)	
H22	0.6408	0.8459	0.1402	0.022*	
C23	0.58692 (9)	0.80824 (16)	0.08526 (9)	0.0200 (4)	
H23	0.5721	0.8672	0.0798	0.024*	
C24	0.56850 (9)	0.73228 (17)	0.05761 (10)	0.0198 (4)	
H24	0.5409	0.7387	0.0328	0.024*	
C25	0.59096 (8)	0.64744 (15)	0.06679 (9)	0.0153 (4)	
H25	0.5780	0.5959	0.0480	0.018*	
C26	0.54766 (8)	0.45919 (16)	0.15431 (8)	0.0162 (4)	
H26	0.5448	0.4377	0.1193	0.019*	
C27	0.50407 (9)	0.4532 (2)	0.18677 (9)	0.0241 (5)	
H27	0.4721	0.4272	0.1744	0.029*	
C28	0.50789 (9)	0.4858 (2)	0.23773 (9)	0.0227 (5)	
H28	0.4784	0.4837	0.2606	0.027*	
C29	0.55529 (9)	0.52141 (19)	0.25454 (9)	0.0214 (5)	
H29	0.5590	0.5435	0.2894	0.026*	
C30	0.59751 (8)	0.52459 (16)	0.22017 (8)	0.0156 (4)	
H30	0.6301	0.5490	0.2320	0.019*	
C31	0.71928 (9)	0.35834 (16)	0.17617 (8)	0.0177 (4)	
H31	0.7389	0.4096	0.1883	0.021*	
C32	0.73252 (10)	0.27212 (18)	0.19426 (9)	0.0222 (5)	
H32	0.7609	0.2642	0.2180	0.027*	
C33	0.70374 (10)	0.19765 (18)	0.17718 (10)	0.0248 (5)	
H33	0.7118	0.1378	0.1893	0.030*	
C34	0.66268 (10)	0.21171 (16)	0.14187 (11)	0.0229 (5)	
H34	0.6425	0.1614	0.1296	0.027*	
C35	0.65162 (9)	0.29982 (16)	0.12486 (9)	0.0169 (4)	
H35	0.6237	0.3092	0.1006	0.020*	
C36	0.77078 (8)	0.51247 (15)	0.08380 (8)	0.0136 (4)	
H36	0.7771	0.5151	0.1205	0.016*	
C37	0.81361 (8)	0.51134 (16)	0.04979 (9)	0.0164 (4)	
H37	0.8486	0.5123	0.0631	0.020*	
C38	0.80443 (8)	0.50876 (16)	−0.00376 (9)	0.0174 (4)	
H38	0.8330	0.5078	−0.0278	0.021*	
C39	0.75289 (9)	0.50755 (16)	−0.02167 (8)	0.0171 (4)	
H39	0.7457	0.5069	−0.0582	0.020*	
C40	0.71206 (8)	0.50729 (15)	0.01433 (8)	0.0137 (4)	
H40	0.6768	0.5052	0.0018	0.016*	
Cl1P	0.63013 (3)	−0.20630 (5)	0.28660 (3)	0.01264 (18)	0.868 (3)
O1P	0.60224 (9)	−0.23420 (16)	0.33334 (8)	0.0262 (5)	0.868 (3)
O2P	0.68283 (8)	−0.1857 (2)	0.30024 (11)	0.0374 (7)	0.868 (3)
O3P	0.62633 (11)	−0.2764 (3)	0.24867 (13)	0.0354 (7)	0.868 (3)
O4P	0.60397 (9)	−0.12621 (15)	0.26743 (10)	0.0370 (5)	0.868 (3)
Cl2P	0.6450 (2)	−0.1831 (4)	0.2710 (2)	0.0221 (12)	0.132 (3)
O5P	0.6630 (6)	−0.2113 (10)	0.3260 (5)	0.0221 (12)	0.132 (3)
O6P	0.6833 (5)	−0.1475 (10)	0.2396 (5)	0.0221 (12)	0.132 (3)
O7P	0.6415 (8)	−0.2796 (17)	0.2504 (9)	0.0221 (12)	0.132 (3)
O8P	0.60397 (9)	−0.12621 (15)	0.26743 (10)	0.0370 (5)	0.132 (3)
Zn1	0.51089 (2)	0.13481 (2)	0.06065 (2)	0.01240 (7)	
Cl1	0.52526 (2)	0.28484 (4)	0.05651 (2)	0.02034 (12)	
Cl2	0.42603 (2)	0.10088 (4)	0.04318 (2)	0.02115 (12)	
Cl3	0.55209 (11)	0.09300 (14)	−0.00286 (9)	0.1300 (9)	
Cl4	0.54386 (3)	0.04804 (5)	0.12572 (2)	0.03005 (14)	
Na1P	0.65188 (10)	−0.00419 (16)	0.19280 (11)	0.0856 (7)	

### Atomic displacement parameters (Å^2^)

**Table:**

	*U*^11^	*U*^22^	*U*^33^	*U*^12^	*U*^13^	*U*^23^
Cr1	0.00589 (14)	0.00984 (15)	0.00688 (14)	−0.00199 (10)	0.00172 (9)	0.00211 (10)
F1	0.0085 (5)	0.0164 (6)	0.0106 (5)	−0.0028 (4)	0.0025 (4)	0.0051 (4)
F2	0.0136 (5)	0.0156 (6)	0.0110 (5)	−0.0050 (4)	0.0066 (4)	0.0024 (4)
N1	0.0108 (7)	0.0129 (8)	0.0111 (7)	0.0001 (6)	−0.0006 (6)	0.0004 (6)
N2	0.0108 (7)	0.0113 (8)	0.0093 (7)	−0.0014 (6)	−0.0008 (6)	0.0016 (6)
N3	0.0088 (7)	0.0130 (8)	0.0079 (7)	−0.0006 (6)	−0.0003 (5)	0.0010 (6)
N4	0.0066 (7)	0.0150 (8)	0.0106 (7)	−0.0008 (6)	0.0008 (5)	0.0003 (6)
C1	0.0266 (11)	0.0125 (10)	0.0173 (9)	−0.0004 (8)	0.0012 (8)	0.0049 (7)
C2	0.0433 (15)	0.0134 (10)	0.0208 (11)	0.0032 (10)	−0.0058 (10)	0.0040 (8)
C3	0.0364 (14)	0.0183 (11)	0.0258 (11)	0.0116 (10)	−0.0128 (10)	−0.0055 (9)
C4	0.0216 (11)	0.0263 (13)	0.0279 (12)	0.0073 (9)	−0.0003 (9)	−0.0106 (10)
C5	0.0152 (9)	0.0196 (11)	0.0192 (10)	−0.0004 (8)	0.0036 (7)	−0.0043 (8)
C6	0.0157 (9)	0.0166 (9)	0.0107 (8)	−0.0003 (7)	−0.0004 (7)	0.0011 (7)
C7	0.0217 (10)	0.0238 (11)	0.0110 (9)	0.0020 (8)	−0.0039 (8)	−0.0011 (8)
C8	0.0183 (10)	0.0188 (10)	0.0179 (10)	0.0003 (8)	−0.0083 (8)	0.0003 (8)
C9	0.0124 (9)	0.0179 (10)	0.0193 (10)	−0.0018 (7)	−0.0036 (7)	0.0026 (8)
C10	0.0108 (8)	0.0169 (9)	0.0121 (8)	−0.0023 (7)	−0.0009 (7)	0.0023 (7)
C11	0.0143 (8)	0.0125 (9)	0.0107 (8)	−0.0023 (7)	−0.0007 (7)	0.0015 (7)
C12	0.0221 (10)	0.0116 (9)	0.0157 (9)	−0.0014 (7)	−0.0031 (7)	0.0010 (7)
C13	0.0216 (10)	0.0156 (10)	0.0157 (9)	0.0058 (8)	−0.0048 (8)	−0.0015 (7)
C14	0.0149 (9)	0.0210 (10)	0.0160 (9)	0.0048 (8)	0.0013 (7)	−0.0020 (8)
C15	0.0115 (8)	0.0174 (10)	0.0122 (8)	0.0003 (7)	0.0023 (7)	0.0005 (7)
C16	0.0096 (9)	0.0503 (16)	0.0177 (10)	−0.0052 (9)	−0.0016 (8)	0.0148 (10)
C17	0.0160 (10)	0.0515 (17)	0.0188 (11)	−0.0053 (10)	−0.0057 (8)	0.0147 (11)
C18	0.0106 (9)	0.0430 (15)	0.0197 (10)	0.0016 (9)	−0.0049 (8)	−0.0003 (10)
C19	0.0085 (10)	0.104 (3)	0.0196 (12)	−0.0125 (14)	−0.0014 (9)	0.0057 (15)
C20	0.0097 (9)	0.067 (2)	0.0133 (10)	−0.0130 (11)	0.0004 (8)	0.0044 (11)
Cr2	0.00645 (14)	0.01014 (15)	0.00675 (14)	−0.00042 (10)	−0.00057 (9)	−0.00142 (10)
F3	0.0122 (5)	0.0140 (6)	0.0091 (5)	−0.0032 (4)	−0.0019 (4)	−0.0029 (4)
F4	0.0099 (5)	0.0185 (6)	0.0107 (5)	−0.0007 (4)	−0.0030 (4)	−0.0032 (4)
N5	0.0089 (7)	0.0115 (8)	0.0104 (7)	−0.0010 (5)	0.0010 (6)	−0.0006 (6)
N6	0.0087 (7)	0.0121 (8)	0.0087 (7)	0.0002 (5)	0.0001 (5)	−0.0011 (5)
N7	0.0129 (7)	0.0142 (8)	0.0108 (7)	0.0039 (6)	0.0027 (6)	0.0009 (6)
N8	0.0084 (7)	0.0135 (8)	0.0093 (7)	0.0004 (6)	0.0001 (5)	−0.0008 (6)
C21	0.0181 (9)	0.0131 (10)	0.0118 (8)	−0.0020 (7)	0.0016 (7)	−0.0024 (7)
C22	0.0269 (11)	0.0132 (10)	0.0159 (9)	−0.0014 (8)	0.0053 (8)	−0.0030 (7)
C23	0.0223 (10)	0.0151 (10)	0.0225 (10)	0.0050 (8)	0.0086 (8)	0.0032 (8)
C24	0.0147 (9)	0.0181 (11)	0.0264 (11)	0.0019 (8)	−0.0014 (8)	0.0056 (8)
C25	0.0135 (9)	0.0147 (10)	0.0178 (9)	−0.0017 (7)	−0.0042 (7)	0.0016 (7)
C26	0.0122 (9)	0.0231 (11)	0.0134 (9)	−0.0065 (7)	0.0014 (7)	−0.0047 (7)
C27	0.0134 (9)	0.0409 (15)	0.0180 (10)	−0.0110 (9)	0.0028 (8)	−0.0065 (10)
C28	0.0137 (10)	0.0383 (14)	0.0160 (10)	−0.0030 (9)	0.0048 (7)	−0.0036 (9)
C29	0.0149 (9)	0.0369 (13)	0.0125 (9)	−0.0013 (9)	0.0033 (7)	−0.0077 (9)
C30	0.0114 (8)	0.0246 (11)	0.0110 (8)	−0.0010 (7)	0.0004 (7)	−0.0056 (8)
C31	0.0187 (10)	0.0213 (11)	0.0133 (9)	0.0069 (8)	0.0002 (7)	0.0007 (7)
C32	0.0254 (11)	0.0253 (12)	0.0160 (9)	0.0129 (9)	0.0025 (8)	0.0037 (8)
C33	0.0292 (12)	0.0207 (11)	0.0244 (11)	0.0121 (9)	0.0109 (9)	0.0073 (9)
C34	0.0245 (11)	0.0129 (10)	0.0312 (12)	0.0022 (8)	0.0087 (9)	0.0006 (9)
C35	0.0154 (9)	0.0139 (10)	0.0214 (10)	0.0021 (7)	0.0051 (7)	0.0000 (7)
C36	0.0094 (8)	0.0194 (10)	0.0121 (8)	0.0003 (7)	−0.0004 (7)	−0.0005 (7)
C37	0.0087 (8)	0.0232 (11)	0.0172 (9)	0.0015 (7)	0.0011 (7)	0.0013 (8)
C38	0.0135 (9)	0.0224 (11)	0.0162 (9)	0.0036 (8)	0.0060 (7)	0.0009 (8)
C39	0.0168 (9)	0.0239 (11)	0.0104 (8)	0.0016 (8)	0.0033 (7)	0.0007 (7)
C40	0.0120 (8)	0.0185 (10)	0.0105 (8)	0.0009 (7)	−0.0001 (7)	0.0001 (7)
Cl1P	0.0113 (3)	0.0140 (3)	0.0127 (3)	0.0026 (2)	−0.0047 (2)	−0.0003 (2)
O1P	0.0341 (11)	0.0252 (11)	0.0193 (9)	−0.0025 (9)	0.0063 (8)	0.0046 (8)
O2P	0.0090 (9)	0.0603 (17)	0.0430 (14)	−0.0026 (10)	−0.0084 (9)	−0.0126 (12)
O3P	0.0316 (15)	0.0470 (16)	0.0276 (12)	0.0024 (15)	−0.0071 (13)	−0.0239 (11)
O4P	0.0288 (10)	0.0346 (12)	0.0476 (13)	0.0188 (9)	0.0081 (9)	0.0216 (10)
Cl2P	0.0194 (14)	0.0263 (14)	0.0205 (14)	−0.0016 (9)	−0.0010 (9)	−0.0038 (9)
O5P	0.0194 (14)	0.0263 (14)	0.0205 (14)	−0.0016 (9)	−0.0010 (9)	−0.0038 (9)
O6P	0.0194 (14)	0.0263 (14)	0.0205 (14)	−0.0016 (9)	−0.0010 (9)	−0.0038 (9)
O7P	0.0194 (14)	0.0263 (14)	0.0205 (14)	−0.0016 (9)	−0.0010 (9)	−0.0038 (9)
O8P	0.0288 (10)	0.0346 (12)	0.0476 (13)	0.0188 (9)	0.0081 (9)	0.0216 (10)
Zn1	0.01514 (13)	0.01067 (13)	0.01138 (12)	−0.00326 (8)	0.00344 (8)	−0.00087 (8)
Cl1	0.0246 (3)	0.0112 (2)	0.0252 (3)	−0.00530 (19)	−0.0023 (2)	−0.00346 (18)
Cl2	0.0145 (2)	0.0141 (2)	0.0348 (3)	−0.00498 (17)	0.0031 (2)	0.0015 (2)
Cl3	0.173 (2)	0.0881 (13)	0.1291 (17)	−0.0183 (13)	0.0785 (16)	−0.0087 (11)
Cl4	0.0477 (4)	0.0215 (3)	0.0209 (3)	0.0029 (3)	−0.0073 (2)	0.0041 (2)
Na1P	0.0897 (16)	0.0696 (14)	0.0974 (17)	−0.0081 (12)	0.0211 (13)	0.0102 (12)

### Geometric parameters (Å, º)

**Table:**

Cr1—F1	1.8532 (12)	N6—C26	1.344 (3)
Cr1—F2	1.8838 (12)	N6—C30	1.345 (2)
Cr1—N4	2.0759 (17)	N7—C35	1.346 (3)
Cr1—N1	2.0902 (18)	N7—C31	1.353 (3)
Cr1—N3	2.0906 (18)	N8—C40	1.349 (2)
Cr1—N2	2.0920 (17)	N8—C36	1.350 (2)
N1—C5	1.343 (3)	C21—C22	1.394 (3)
N1—C1	1.349 (3)	C21—H21	0.9500
N2—C6	1.347 (3)	C22—C23	1.382 (3)
N2—C10	1.348 (2)	C22—H22	0.9500
N3—C11	1.348 (3)	C23—C24	1.395 (4)
N3—C15	1.348 (2)	C23—H23	0.9500
N4—C20	1.334 (3)	C24—C25	1.384 (3)
N4—C16	1.339 (3)	C24—H24	0.9500
C1—C2	1.386 (3)	C25—H25	0.9500
C1—H1	0.9500	C26—C27	1.385 (3)
C2—C3	1.384 (4)	C26—H26	0.9500
C2—H2	0.9500	C27—C28	1.388 (3)
C3—C4	1.392 (4)	C27—H27	0.9500
C3—H3	0.9500	C28—C29	1.380 (3)
C4—C5	1.382 (3)	C28—H28	0.9500
C4—H4	0.9500	C29—C30	1.386 (3)
C5—H5	0.9500	C29—H29	0.9500
C6—C7	1.387 (3)	C30—H30	0.9500
C6—H6	0.9500	C31—C32	1.382 (3)
C7—C8	1.391 (3)	C31—H31	0.9500
C7—H7	0.9500	C32—C33	1.381 (4)
C8—C9	1.388 (3)	C32—H32	0.9500
C8—H8	0.9500	C33—C34	1.393 (4)
C9—C10	1.386 (3)	C33—H33	0.9500
C9—H9	0.9500	C34—C35	1.386 (3)
C10—H10	0.9500	C34—H34	0.9500
C11—C12	1.393 (3)	C35—H35	0.9500
C11—H11	0.9500	C36—C37	1.391 (3)
C12—C13	1.388 (3)	C36—H36	0.9500
C12—H12	0.9500	C37—C38	1.386 (3)
C13—C14	1.387 (3)	C37—H37	0.9500
C13—H13	0.9500	C38—C39	1.387 (3)
C14—C15	1.385 (3)	C38—H38	0.9500
C14—H14	0.9500	C39—C40	1.385 (3)
C15—H15	0.9500	C39—H39	0.9500
C16—C17	1.382 (3)	C40—H40	0.9500
C16—H16	0.9500	Cl1P—O3P	1.412 (3)
C17—C18	1.372 (3)	Cl1P—O2P	1.415 (2)
C17—H17	0.9500	Cl1P—O4P	1.431 (2)
C18—C19	1.379 (4)	Cl1P—O1P	1.445 (2)
C18—H18	0.9500	O4P—Na1P	2.877 (3)
C19—C20	1.388 (3)	Cl2P—O6P	1.362 (15)
C19—H19	0.9500	Cl2P—O7P	1.51 (3)
C20—H20	0.9500	Cl2P—O5P	1.531 (15)
Cr2—F3	1.8552 (12)	Cl2P—Na1P	3.291 (8)
Cr2—F4	1.8634 (12)	O6P—Na1P	2.537 (15)
Cr2—N7	2.0769 (18)	Zn1—Cl3	2.0228 (19)
Cr2—N6	2.0801 (17)	Zn1—Cl1	2.2232 (7)
Cr2—N5	2.0868 (18)	Zn1—Cl4	2.2499 (7)
Cr2—N8	2.0986 (17)	Zn1—Cl2	2.2558 (7)
N5—C21	1.342 (3)	Cl4—Na1P	3.322 (3)
N5—C25	1.349 (3)		
			
F1—Cr1—F2	178.47 (6)	N6—Cr2—N8	178.11 (7)
F1—Cr1—N4	89.83 (6)	N5—Cr2—N8	93.92 (7)
F2—Cr1—N4	91.70 (6)	C21—N5—C25	118.28 (19)
F1—Cr1—N1	90.50 (6)	C21—N5—Cr2	120.52 (14)
F2—Cr1—N1	89.61 (6)	C25—N5—Cr2	120.81 (14)
N4—Cr1—N1	87.93 (7)	C26—N6—C30	118.39 (18)
F1—Cr1—N3	89.90 (6)	C26—N6—Cr2	120.70 (14)
F2—Cr1—N3	90.04 (6)	C30—N6—Cr2	120.90 (14)
N4—Cr1—N3	90.12 (7)	C35—N7—C31	118.73 (19)
N1—Cr1—N3	178.00 (7)	C35—N7—Cr2	119.82 (15)
F1—Cr1—N2	88.25 (6)	C31—N7—Cr2	121.30 (15)
F2—Cr1—N2	90.22 (6)	C40—N8—C36	118.38 (17)
N4—Cr1—N2	176.95 (7)	C40—N8—Cr2	120.77 (14)
N1—Cr1—N2	89.72 (7)	C36—N8—Cr2	120.79 (13)
N3—Cr1—N2	92.25 (7)	N5—C21—C22	122.2 (2)
C5—N1—C1	118.3 (2)	N5—C21—H21	118.9
C5—N1—Cr1	121.84 (15)	C22—C21—H21	118.9
C1—N1—Cr1	119.77 (15)	C23—C22—C21	119.5 (2)
C6—N2—C10	118.25 (17)	C23—C22—H22	120.3
C6—N2—Cr1	121.93 (14)	C21—C22—H22	120.3
C10—N2—Cr1	119.74 (13)	C22—C23—C24	118.3 (2)
C11—N3—C15	117.97 (18)	C22—C23—H23	120.9
C11—N3—Cr1	121.69 (14)	C24—C23—H23	120.9
C15—N3—Cr1	120.33 (14)	C25—C24—C23	119.2 (2)
C20—N4—C16	117.59 (19)	C25—C24—H24	120.4
C20—N4—Cr1	122.37 (15)	C23—C24—H24	120.4
C16—N4—Cr1	119.92 (14)	N5—C25—C24	122.5 (2)
N1—C1—C2	122.4 (2)	N5—C25—H25	118.7
N1—C1—H1	118.8	C24—C25—H25	118.7
C2—C1—H1	118.8	N6—C26—C27	122.5 (2)
C3—C2—C1	119.1 (2)	N6—C26—H26	118.8
C3—C2—H2	120.4	C27—C26—H26	118.8
C1—C2—H2	120.4	C26—C27—C28	118.8 (2)
C2—C3—C4	118.4 (2)	C26—C27—H27	120.6
C2—C3—H3	120.8	C28—C27—H27	120.6
C4—C3—H3	120.8	C29—C28—C27	118.8 (2)
C5—C4—C3	119.3 (2)	C29—C28—H28	120.6
C5—C4—H4	120.3	C27—C28—H28	120.6
C3—C4—H4	120.3	C28—C29—C30	119.4 (2)
N1—C5—C4	122.4 (2)	C28—C29—H29	120.3
N1—C5—H5	118.8	C30—C29—H29	120.3
C4—C5—H5	118.8	N6—C30—C29	122.08 (19)
N2—C6—C7	122.3 (2)	N6—C30—H30	119.0
N2—C6—H6	118.8	C29—C30—H30	119.0
C7—C6—H6	118.8	N7—C31—C32	122.4 (2)
C6—C7—C8	119.1 (2)	N7—C31—H31	118.8
C6—C7—H7	120.5	C32—C31—H31	118.8
C8—C7—H7	120.5	C33—C32—C31	118.9 (2)
C9—C8—C7	118.90 (19)	C33—C32—H32	120.6
C9—C8—H8	120.5	C31—C32—H32	120.6
C7—C8—H8	120.5	C32—C33—C34	119.0 (2)
C10—C9—C8	118.8 (2)	C32—C33—H33	120.5
C10—C9—H9	120.6	C34—C33—H33	120.5
C8—C9—H9	120.6	C35—C34—C33	119.4 (2)
N2—C10—C9	122.72 (19)	C35—C34—H34	120.3
N2—C10—H10	118.6	C33—C34—H34	120.3
C9—C10—H10	118.6	N7—C35—C34	121.6 (2)
N3—C11—C12	122.42 (19)	N7—C35—H35	119.2
N3—C11—H11	118.8	C34—C35—H35	119.2
C12—C11—H11	118.8	N8—C36—C37	122.27 (19)
C13—C12—C11	119.1 (2)	N8—C36—H36	118.9
C13—C12—H12	120.5	C37—C36—H36	118.9
C11—C12—H12	120.5	C38—C37—C36	118.91 (19)
C14—C13—C12	118.6 (2)	C38—C37—H37	120.5
C14—C13—H13	120.7	C36—C37—H37	120.5
C12—C13—H13	120.7	C37—C38—C39	118.92 (19)
C15—C14—C13	119.2 (2)	C37—C38—H38	120.5
C15—C14—H14	120.4	C39—C38—H38	120.5
C13—C14—H14	120.4	C40—C39—C38	119.2 (2)
N3—C15—C14	122.7 (2)	C40—C39—H39	120.4
N3—C15—H15	118.6	C38—C39—H39	120.4
C14—C15—H15	118.6	N8—C40—C39	122.26 (19)
N4—C16—C17	123.1 (2)	N8—C40—H40	118.9
N4—C16—H16	118.5	C39—C40—H40	118.9
C17—C16—H16	118.5	O3P—Cl1P—O2P	112.79 (16)
C18—C17—C16	119.1 (2)	O3P—Cl1P—O4P	109.07 (18)
C18—C17—H17	120.5	O2P—Cl1P—O4P	110.45 (17)
C16—C17—H17	120.5	O3P—Cl1P—O1P	109.10 (19)
C17—C18—C19	118.4 (2)	O2P—Cl1P—O1P	108.70 (16)
C17—C18—H18	120.8	O4P—Cl1P—O1P	106.53 (13)
C19—C18—H18	120.8	Cl1P—O4P—Na1P	122.39 (13)
C18—C19—C20	119.3 (2)	O6P—Cl2P—O7P	101.2 (11)
C18—C19—H19	120.3	O6P—Cl2P—O5P	115.5 (9)
C20—C19—H19	120.3	O7P—Cl2P—O5P	94.8 (11)
N4—C20—C19	122.5 (2)	O6P—Cl2P—Na1P	45.8 (6)
N4—C20—H20	118.7	O7P—Cl2P—Na1P	122.3 (10)
C19—C20—H20	118.7	O5P—Cl2P—Na1P	138.9 (6)
F3—Cr2—F4	177.55 (6)	Cl2P—O6P—Na1P	111.5 (8)
F3—Cr2—N7	90.85 (7)	Cl3—Zn1—Cl1	100.05 (6)
F4—Cr2—N7	89.38 (7)	Cl3—Zn1—Cl4	103.21 (9)
F3—Cr2—N6	90.48 (6)	Cl1—Zn1—Cl4	121.91 (3)
F4—Cr2—N6	91.96 (6)	Cl3—Zn1—Cl2	105.65 (9)
N7—Cr2—N6	88.54 (7)	Cl1—Zn1—Cl2	111.33 (2)
F3—Cr2—N5	89.46 (6)	Cl4—Zn1—Cl2	112.19 (3)
F4—Cr2—N5	90.51 (6)	Zn1—Cl4—Na1P	144.75 (5)
N7—Cr2—N5	175.43 (7)	O6P—Na1P—Cl2P	22.6 (3)
N6—Cr2—N5	86.89 (7)	O6P—Na1P—Cl4	133.7 (3)
F3—Cr2—N8	87.82 (6)	O4P—Na1P—Cl4	97.68 (9)
F4—Cr2—N8	89.74 (6)	Cl2P—Na1P—Cl4	116.80 (12)
N7—Cr2—N8	90.65 (7)		
			
C5—N1—C1—C2	1.1 (3)	C21—C22—C23—C24	−0.1 (3)
Cr1—N1—C1—C2	−175.75 (19)	C22—C23—C24—C25	0.3 (3)
N1—C1—C2—C3	1.4 (4)	C21—N5—C25—C24	0.6 (3)
C1—C2—C3—C4	−2.8 (4)	Cr2—N5—C25—C24	173.43 (17)
C2—C3—C4—C5	1.9 (4)	C23—C24—C25—N5	−0.5 (3)
C1—N1—C5—C4	−2.1 (3)	C30—N6—C26—C27	−0.1 (4)
Cr1—N1—C5—C4	174.69 (18)	Cr2—N6—C26—C27	−178.8 (2)
C3—C4—C5—N1	0.6 (4)	N6—C26—C27—C28	1.0 (4)
C10—N2—C6—C7	−0.7 (3)	C26—C27—C28—C29	−1.3 (4)
Cr1—N2—C6—C7	176.06 (17)	C27—C28—C29—C30	0.7 (4)
N2—C6—C7—C8	0.1 (3)	C26—N6—C30—C29	−0.5 (3)
C6—C7—C8—C9	0.7 (3)	Cr2—N6—C30—C29	178.14 (19)
C7—C8—C9—C10	−0.8 (3)	C28—C29—C30—N6	0.2 (4)
C6—N2—C10—C9	0.5 (3)	C35—N7—C31—C32	0.2 (3)
Cr1—N2—C10—C9	−176.32 (17)	Cr2—N7—C31—C32	175.81 (17)
C8—C9—C10—N2	0.3 (3)	N7—C31—C32—C33	−0.7 (3)
C15—N3—C11—C12	−1.5 (3)	C31—C32—C33—C34	0.7 (3)
Cr1—N3—C11—C12	177.33 (15)	C32—C33—C34—C35	−0.2 (4)
N3—C11—C12—C13	0.0 (3)	C31—N7—C35—C34	0.3 (3)
C11—C12—C13—C14	0.9 (3)	Cr2—N7—C35—C34	−175.35 (17)
C12—C13—C14—C15	−0.4 (3)	C33—C34—C35—N7	−0.4 (3)
C11—N3—C15—C14	2.1 (3)	C40—N8—C36—C37	−0.8 (3)
Cr1—N3—C15—C14	−176.77 (16)	Cr2—N8—C36—C37	176.29 (17)
C13—C14—C15—N3	−1.2 (3)	N8—C36—C37—C38	0.9 (3)
C20—N4—C16—C17	−0.5 (4)	C36—C37—C38—C39	0.1 (3)
Cr1—N4—C16—C17	175.7 (2)	C37—C38—C39—C40	−1.2 (3)
N4—C16—C17—C18	−0.4 (5)	C36—N8—C40—C39	−0.3 (3)
C16—C17—C18—C19	1.1 (5)	Cr2—N8—C40—C39	−177.39 (17)
C17—C18—C19—C20	−1.0 (6)	C38—C39—C40—N8	1.3 (3)
C16—N4—C20—C19	0.6 (5)	O3P—Cl1P—O4P—Na1P	−79.1 (2)
Cr1—N4—C20—C19	−175.5 (3)	O2P—Cl1P—O4P—Na1P	45.3 (2)
C18—C19—C20—N4	0.1 (6)	O1P—Cl1P—O4P—Na1P	163.23 (16)
C25—N5—C21—C22	−0.5 (3)	O7P—Cl2P—O6P—Na1P	124.6 (10)
Cr2—N5—C21—C22	−173.30 (16)	O5P—Cl2P—O6P—Na1P	−134.5 (8)
N5—C21—C22—C23	0.2 (3)		

### Hydrogen-bond geometry (Å, º)

**Table:**

*D*—H···*A*	*D*—H	H···*A*	*D*···*A*	*D*—H···*A*
C2—H2···O5*P*^i^	0.95	2.31	3.237 (14)	165
C10—H10···O2*P*^ii^	0.95	2.49	3.352 (3)	151

Symmetry codes: (i) −*x*+1, *y*+1/2, −*z*+1/2; (ii) *x*−1/2, *y*+1, −*z*+1/2.
